# Pulmonary carcinosarcoma showing an obvious response to pazopanib: a case report

**DOI:** 10.1186/s12890-018-0757-7

**Published:** 2018-12-12

**Authors:** Azusa Tanimoto, Shinji Takeuchi, Hiroshi Kotani, Kaname Yamashita, Tadaaki Yamada, Koushiro Ohtsubo, Hiromichi Ebi, Hiroko Ikeda, Seiji Yano

**Affiliations:** 1 Division of Medical Oncology, Cancer Research Institute, Kanazawa University, 13-1, Takara-machi, Kanazawa, Ishikawa 920-0934 Japan; 2 Department of Pulmonary Medicine, Graduate School of Medical Science, Kyoto Prefectural University of Medicine, Kyoto, Japan; 3 Division of Pathology, Kanazawa University Hospital, Kanazawa, Japan

**Keywords:** Pulmonary carcinosarcoma, Pazopanib, Thrombocytopenia, VEGFR

## Abstract

**Background:**

Pulmonary carcinosarcoma (PCS) is a rare primary lung malignancy and has a poor prognosis among lung tumor histological subtypes. However, an appropriate treatment strategy has not been developed for unresectable PCS.

**Case presentation:**

A 65-year-old man who was diagnosed with PCS was treated by surgical removal of the primary lung lesion, followed by six cycles of adjuvant chemotherapy with cisplatin plus irinotecan. Following the chemotherapy, he experienced a relapse with brain metastasis, which induced the rapid onset of left leg paralysis. Radical surgical resection and stereotactic radiosurgery to the resection cavity were performed. However, meningeal dissemination and new lung metastases occurred after a year and half. To control these multiple metastatic lesions, the patient was treated with the multiple kinase inhibitor pazopanib. No change was observed in the meningeal dissemination, while the metastatic lung lesions were prominently reduced in size following treatment with pazopanib. Consequently, the patient showed a partial response to pazopanib treatment, although the dose of pazopanib was reduced by half as a result of thrombocytopenia.

**Conclusion:**

This is the first report of metastatic PCS showing an evident therapeutic response to tumor-targeted therapy. We suggest that pazopanib may be a therapeutic option for patients with metastatic PCS.

## Background

Pulmonary carcinosarcoma (PCS), which accounts for only 0.3% of all lung tumors, is a rare histological subtype of non-small lung cancer (NSCLC) [1]. PCS is defined by the presence of epithelial elements (squamous or adenocarcinoma) combined with sarcomatous elements such as rhabdomyosarcoma, osteosarcoma, or chondrosarcoma [2]. A few studies have demonstrated the presence of common chromosomal abnormalities in the epithelial and sarcomatous components of PCS [3, 4], suggesting a monoclonal origin of PCS tumors. Standard therapy for PCS has not yet been established owing to the low incidence of this type of tumor. Pazopanib, a multi-targeted tyrosine kinase inhibitor against the proto-oncogene c-Kit (c-KIT), platelet-derived growth factor receptor (PDGFR), fibroblast growth factor receptor (FGFR), and vascular endothelial growth factor receptor (VEGFR), has been reported to show beneficial outcomes in patients with metastatic non-adipocytic soft-tissue sarcoma and renal cell carcinoma, and superior safety and quality-of-life profiles compared with sunitinib, which is a similar targeted drug [5, 6]. Here, we report the first case of metastatic PCS that showed a remarkable response to pazopanib despite the necessity of dose reduction owing to thrombocytopenia.

## Case report

**Fig. 1:**
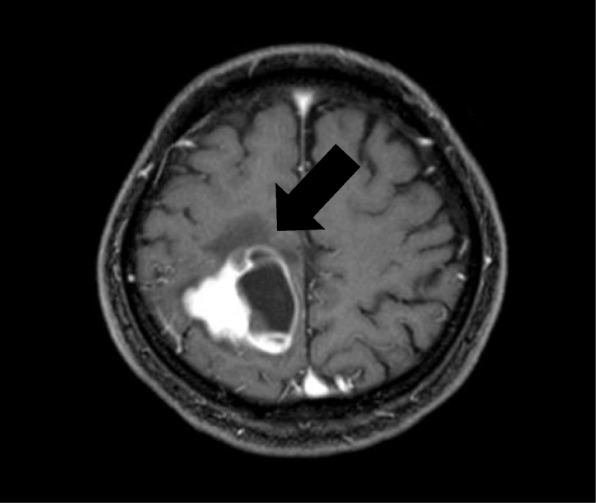
Brain magnetic resonance imaging (MRI) reveals a cerebral ring-enhancing lesion with hemorrhage (arrow)

**Fig. 2:**
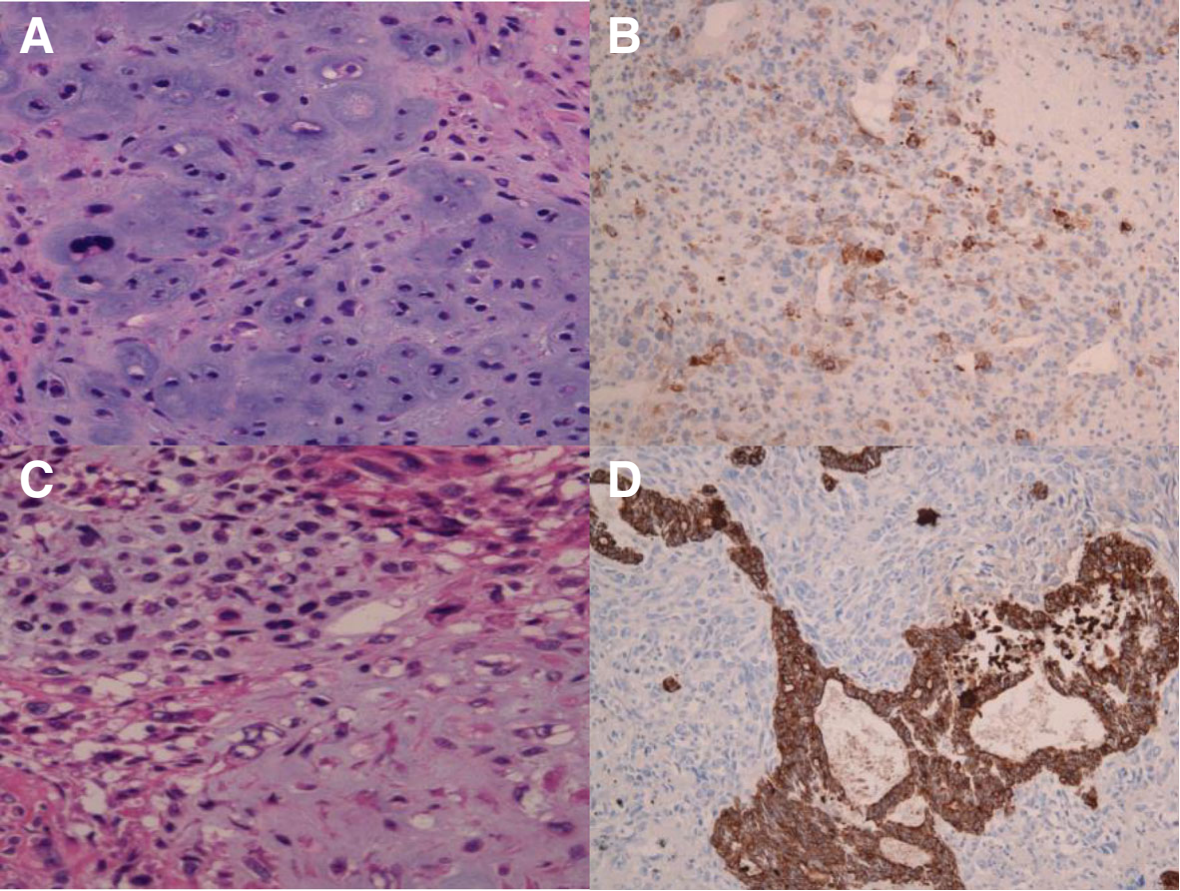
Histological findings: Photomicrograph of resected specimen from brain metastases demonstrates cartilage compositions (**a**) (hematoxylin and eosin [HE] staining, × 100) and epithelial tumors (**b**) (cytokeratin AE1/AE3 staining, × 100). **c**, **d** Photomicrograph of HE and cytokeratin AE1/AE3 stained specimen demonstrates findings conformable with resected specimen from primary lung lesion (magnification, × 100)

**Fig. 3:**
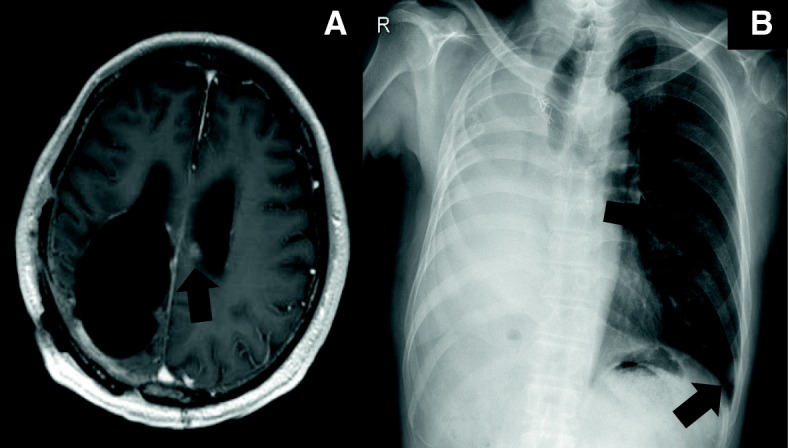
**a** Brain magnetic resonance imaging (MRI) shows meningeal dissemination in left lateral ventricle (arrow). **b** Chest x-ray scan shows two nodule shadows in left lower lung field (arrow)

**Fig. 4:**
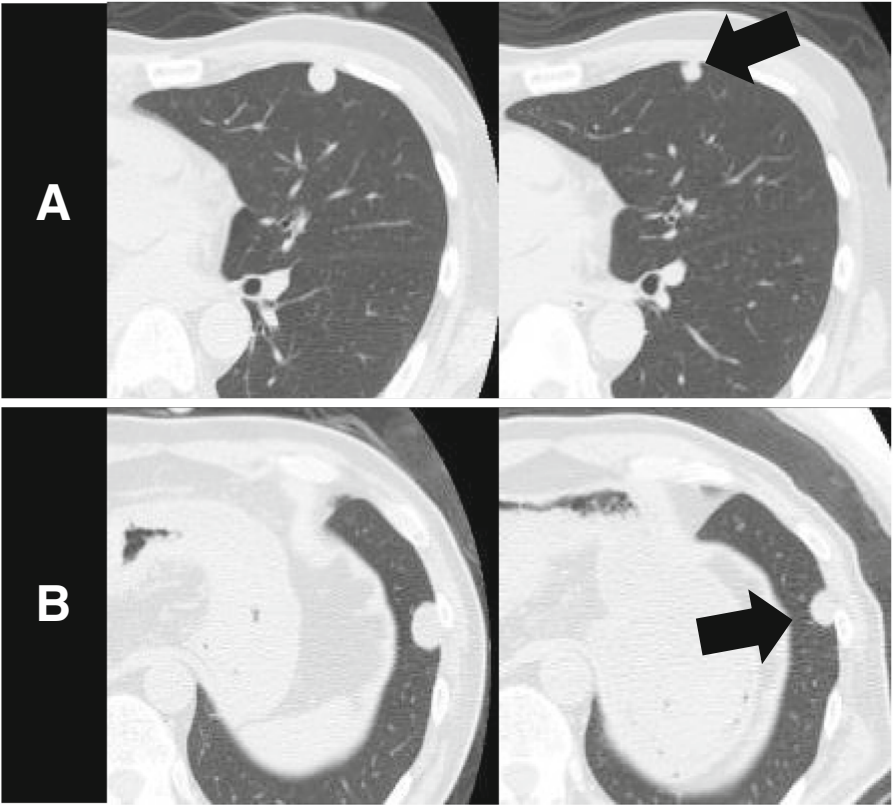
Computed tomography (CT) image illustrates two nodules in left upper lobe (**a**) and lower lobe (**b**) prior to treatment. The size of the nodules (arrow) was reduced following treatment (right panel)

A 65-year-old man who never smoked and had consulted a local hospital 3 years earlier presented to our hospital, where he was diagnosed with PCS and treated with right pneumonectomy, followed by six cycles of adjuvant chemotherapy with cisplatin plus irinotecan, because the pTNM stage was pT3N1M0 stage IIIA according to the 7 th lung cancer TNM classification. One year later, he experienced the rapid onset of left lower extremity paralysis, and brain gadolinium contrast-enhanced magnetic resonance imaging (MRI) performed in our hospital revealed a new brain mass with active bleeding in the right parietal lobe (Fig. 1). The patient was treated with surgical resection, followed by stereotactic radiosurgery to the resection cavity. Immunohistochemical analysis of resected tissue samples revealed sarcomatous tumors composed of spindle cells and cartilage and epithelial tumors expressing cytokeratin AE1/AE3 (Fig. 2a, b). These histological findings were quite similar to those observed with tissue from pneumonectomy specimens (Fig. 2c, d). Therefore, we made a definitive histological diagnosis of metastatic PCS on the brain tumor specimens. A year and a half after this operation, brain gadolinium contrast-enhanced MRI revealed meningeal dissemination (Fig. 3a), and chest radiography identified two nodules in the left lower lung field (Fig. 3b) which were diagnosed with lung recurrence although the lesions were not verified histologically for fear of fatal iatrogenic pneumothorax because of right pneumonectomy. The patient began treatment for PCS with pazopanib (Votrient®, GlaxoSmithKline, Uxbridge, Middlesex, UK) 800 mg orally once a day on the basis of the histological diagnosis because pazopanib has been approved for the treatment of soft tissue sarcoma in Japan. Two weeks after treatment initiation, the patient was withdrawn from the drug because his platelet count was reduced to 60,000/μL. Since the platelet count recovered to above the lower limit of normal 2 weeks later, he was permitted to resume pazopanib at 400 mg orally once a day. Although a brain MRI scan showed no change in the meningeal dissemination, a chest and abdominal computed tomography scan demonstrated a reduction in size of 60% in the lung metastatic lesions of PCS according to Response Evaluation Criteria In Solid Tumors (RECIST) version 1.1 after 2 months of treatment (Fig. 4a-d). His quality of life during the pazopanib treatment had kept adequate because there was little adverse event by pazopanib except thrombocytopenia. 6 months later, the patient showed consciousness disorder due to the progression of the meningeal dissemination and stopped taking pazopanib despite the lung metastatic lesions kept shrinking.

## Discussion and conclusions

Complete surgical resection is the first choice for standard treatment for PCS, if possible. On the other hand, effective systemic chemotherapy in patients with inoperable PCS has not yet been developed [7] although the prognosis of PCS is poorer than that of other types of NSCLC [8]. In the present report, the multiple kinase inhibitor pazopanib showed an obvious effect in a case of metastatic PCS.

Several papers have reported that the prognosis of patients with PCS was poor, with a median survival of approximately 1 year [9, 10]. Although a few reports showed that doxorubicin-based regimens had some effect on metastatic PCS [1, 11], standard chemotherapy for metastatic PCS has not yet been developed [12]. Therefore, pazopanib has the potential to become one of the new treatment options for inoperable PCS.

In-frame deletions at exon 19 in the *EGFR* gene in both the carcinomatous component and the sarcomatous component have been detected in resected PCS specimens [13], indicating the potential to identify oncogenic driver mutations for targeting in PCS tumors. We performed mutational analysis of *KIT Proto-Oncogene Receptor Tyrosine Kinase* (*KIT*) and *Platelet-Derived Growth Factor Receptor-A* (*PDGFRA*) genes in the present case, because pazopanib has inhibitory activity on PDGFR-α/β and c-Kit [14]. We found no active mutations of these genes, suggesting that the tumor regression in the present case might be attributable to inhibition by pazopanib of VEGFR instead of that of KIT and PDGFR. Previous research demonstrated that VEGF, but not c-KIT, epidermal growth factor receptor (EGFR), or human epidermal growth factor receptor 2 (HER-2), was strongly expressed in both the carcinomatous component and the sarcomatous component in 30 cases of uterine carcinosarcoma [15]. Clinical analysis revealed that increased VEGF and VEGFR-3 expression indicated poor survival in uterine and ovarian carcinosarcomas from analysis of 25 patient samples [16]. Therefore, it would be valuable if the expression of VEGF and VEGFR in PCS tumors could be surveyed, which could indicate the possibility of a novel treatment strategy via the VEGF/VEGFR axis in PCS. Further clinical study is needed to elucidate the crucial signals for PCS.

In the present case, the pulmonary metastatic lesions from PCS showed a marked reduction in size in response to pazopanib treatment. This is the first case of PCS demonstrating a good clinical response to molecular targeted therapy. Although a phase II clinical study found that the response rate to pazopanib in uterine carcinosarcoma was 0% (0/19) [17], another study reported the first cases of positive clinical response to pazopanib in patients with uterine carcinosarcoma and ovarian carcinosarcoma [18].

These findings would indicate the heterogeneity of dominant survival signals in carcinosarcoma regardless of the primary lesions. Therefore, the attempt should be made to identify a predictive biomarker, such as VEGF/VEGFR, for response to certain specific drugs in types of carcinosarcoma including PCS.

We have reported the case of a patient with PCS who showed a good response to pazopanib, albeit with dose reduction by half owing to adverse effects. Pazopanib may have potential in the treatment of PCS. Although PCS is extremely rare, continued study of the clinical benefits of pazopanib may aid in the development of a new therapeutic strategy for patients with metastatic PCS.

## Abbreviations

c-KIT: Proto-oncogene c-Kit
PCS: Pulmonary carcinosarcoma
PDGFR: Platelet-derived growth factor receptor
VEGFR: Vascular endothelial growth factor receptor
